# Ultrasonographic whirlpool sign in ovarian torsion with dual mature teratomas: A case report and literature review

**DOI:** 10.3892/mi.2026.322

**Published:** 2026-05-12

**Authors:** Fani-Niki Varra, Apostolos Fasoulopoulos, Eleni Spathi, Ourania Tzirou, Viktoria-Konstantina Varra, Michail Varras

**Affiliations:** 1 Department of Pharmacy, School of Health Sciences, Frederick University, Nicosia 1036, Cyprus; 2 Medical School, Democritus University of Thrace, 68100 Alexandroupolis, Greece; 3 Fourth Department of Obstetrics and Gynecology, ‘Elena Venizelou’ General and Maternity Hospital, 11521 Athens, Greece; 4 Department of Ultrasound, ‘Elena Venizelou’ General and Maternity Hospital, 11521 Athens, Greece; 5 School of Health Sciences, National and Kapodistrian University of Athens, 15771 Athens, Greece; 6 Division of Aesthetics and Cosmetic Science, Department of Biomedical Sciences, School of Health and Welfare Sciences, University of West Attica, 12243 Athens, Greece

**Keywords:** adnexal torsion, ovarian torsion, whirlpool sign, multiple, dual, mature teratoma, dermoid cyst, single ovary, ultrasonography

## Abstract

Adnexal torsion is a gynecological emergency resulting from the partial or complete rotation of the ovary, often involving the fallopian tube, around its vascular pedicle. Prompt diagnosis is crucial to prevent ischemic injury and preserve ovarian function. Mature cystic ovarian teratomas are common benign neoplasms and represent a frequent cause of torsion due to their size, mobility and mass effect. The present case report describes the case of a 36-year-old woman (gravida 2, para 2) who presented several hours following the sudden onset of right lower abdominal pain, initially accompanied by nausea, both of which had resolved by the time of evaluation. A transvaginal ultrasonography revealed a right ovarian mass measuring 6.13x5.20 cm, containing two mature cystic teratomas. Gray-scale and color Doppler imaging clearly demonstrated the characteristic whirlpool sign, indicating torsion of the adnexal vascular pedicle. An emergency laparoscopy revealed the quadruple torsion of the adnexa (fallopian tube and ovary), and a right salpingo-oophorectomy was performed, as the patient had completed her family. The post-operative course of the patient was uneventful. A post-operative pathological examination confirmed the torsion of mature ovarian teratomas with associated hemorrhagic necrosis. On the whole, the present study demonstrates that the ultrasonographic whirlpool sign is a highly specific pre-operative marker for adnexal torsion, particularly in cases with multiple mature cystic teratomas. Early recognition facilitates prompt surgical intervention, minimizes ischemic injury, and may preserve ovarian function. Incorporating the focused assessment of the whirlpool sign into routine ultrasonography for acute pelvic pain enhances diagnostic accuracy and guides timely operative management.

## Introduction

Adnexal torsion in female patients may present as isolated torsion of the ovary, the fallopian tube, or both structures concurrently (1-3). It is most commonly associated with functional ovarian cysts, ovarian tumors-including mature cystic teratomas, cystadenomas, and malignant masses, or hydrosalpinx, which increase the risk of torsion through their size and mass effect on the ovarian pedicle (1-6). Additionally, paraovarian or paratubal masses or cysts may function as mechanical lead points, further predisposing to torsion (1-3). Adnexal torsion accounts for ~2.7-3% of gynecological emergencies and can occur across all age groups, from premenarchal to postmenopausal, with the majority of cases occurring in women of reproductive age (7-9). Ovarian torsion around its vascular pedicle initially impairs venous and lymphatic outflow, causing congestion and stromal edema (10). If not treated promptly, it can progress to arterial obstruction, leading to ischemia and the possible loss of ovarian viability (10). Clinically, adnexal torsion most commonly presents with acute-onset, unilateral lower abdominal or pelvic pain, frequently accompanied by nausea and vomiting (11). In exceedingly rare circumstances, hemoperitoneum may develop, particularly when torsion involves a solid ovarian mass such as a dysgerminoma (12). This can result from passive venous congestion, leading to the rupture of a superficial tumoral vessel and spontaneous intra-abdominal hemorrhage (12). Despite these features, the clinical manifestations of adnexal torsion are often nonspecific and may mimic other acute abdominal or gynecologic conditions, leading to potential diagnostic delay (11). Consequently, this non-specific presentation underscores the importance of imaging for accurate diagnosis, particularly in settings where prompt surgical intervention is essential to preserve ovarian function (10,13,14).

Pelvic and transvaginal ultrasonography remains the first-line imaging modality for suspected adnexal torsion, owing to its accessibility, real-time evaluation and cost-effectiveness (15,16). Gray-scale ultrasound can identify features suggestive of torsion, including asymmetric ovarian enlargement, stromal edema with peripheral follicle displacement, adnexal masses and free pelvic fluid, whereas color or power Doppler ultrasound can identify alterations in vascular flow patterns (1,17). Nevertheless, these indirect signs may be inconsistent, particularly in partial, intermittent, or early torsion, where Doppler flow abnormalities may be absent (10,14). Normal or preserved blood flow does not exclude torsion and may reflect residual arterial perfusion, dual ovarian blood supply, or transient detorsion (10,14). To overcome these limitations, the whirlpool sign has emerged as a specific ultrasonographic marker that directly visualizes the twisted vascular pedicle. The whirlpool sign is identified when the transducer exhibits coiled ovarian vessels, including the utero-ovarian and infundibulopelvic ligaments, arranged concentrically (14). These structures appear to spiral toward the affected adnexa (14). On color Doppler imaging, this appears as concentric circular or spiral vascular flow patterns, representing a direct indicator of torsion (14).

The present case report aimed to highlight the diagnostic value of the whirlpool sign in the timely identification of adnexal torsion associated with a mature ovarian teratoma, a challenging yet clinically significant emergency. By documenting the characteristic gray-scale and color Doppler features of the whirlpool sign, the authors aimed to raise awareness among obstetrician-gynecologists, radiologists and sonographers. Early recognition facilitates prompt surgical intervention, which can preserve ovarian function and fertility in reproductive-aged women. Furthermore, the present case report underscores the importance of meticulous scanning technique and focused evaluation of the adnexal pedicle during ultrasound examination in patients presenting with acute pelvic pain. Ultimately, the case described herein reinforces the whirlpool sign as a critical component of the pre-operative diagnostic framework for adnexal torsion and advocates for its routine assessment in suspected cases.

## Case report

A 36-year-old woman (gravida 2, para 2) with a history of two cesarean sections and one spontaneous abortion presented to the Emergency Department of the ‘Elena Venizelou’ General and Maternity Hospital, Athens, Greece several hours following the sudden onset of right lower abdominal pain, initially accompanied by nausea, both of which had resolved by the time of evaluation. Upon examination, the patient was found to be in a moderate general condition, cooperative, oriented and hemodynamically stable. Upon a physical examination, the lower abdomen was found to be soft with mild tenderness in the right lower quadrant. Uterine tone was normal, and no vaginal bleeding was observed. A bimanual gynecological examination revealed a large, tender mass palpable in the posterior pouch of Douglas.

**Figure 1:**
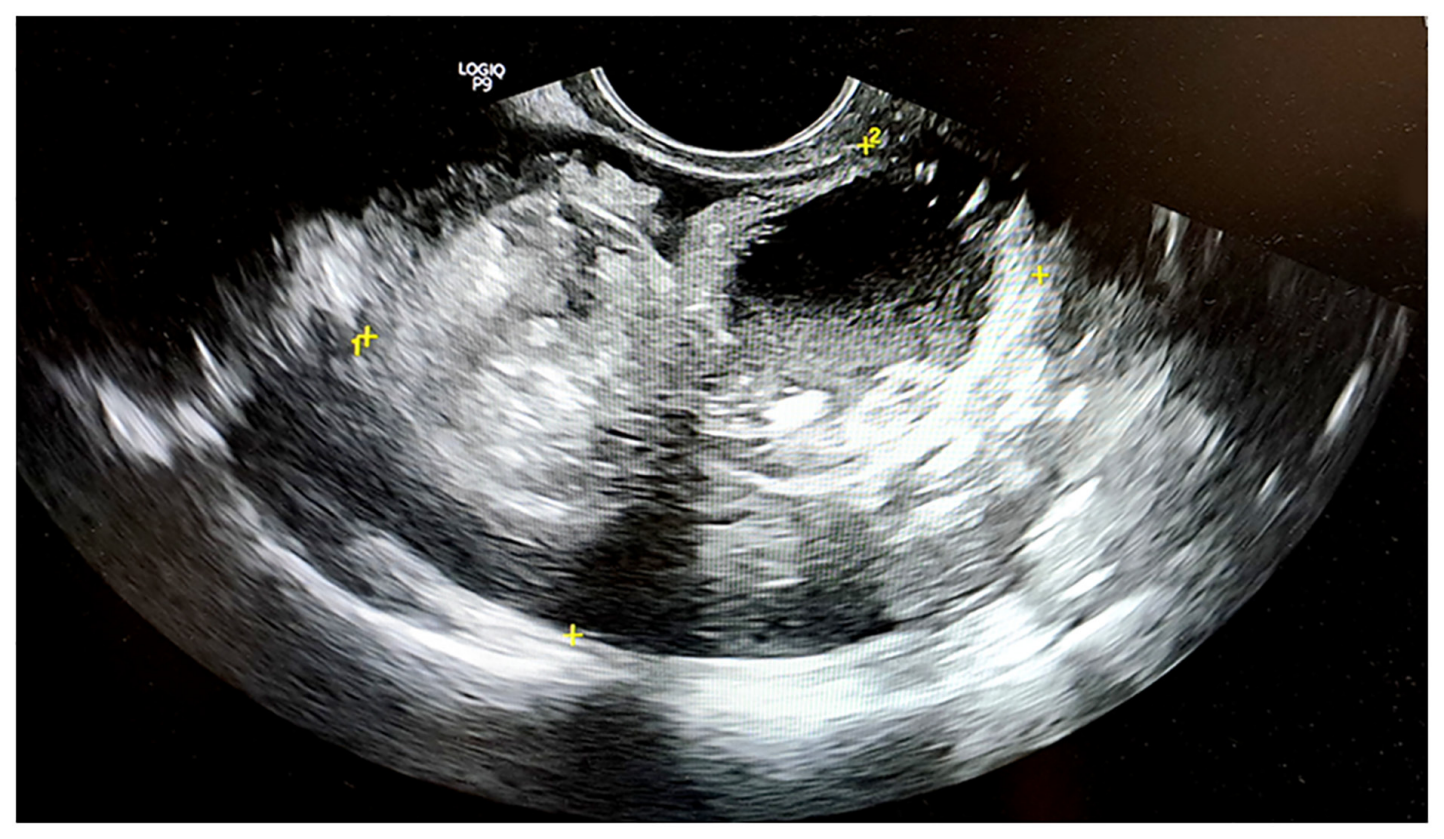
Transvaginal ultrasonography illustrating a bilocular mass within the posterior pouch of Douglas with mixed echogenicity, comprising solid, cystic and fat components, measuring 6.13x5.20 cm; these findings are suggestive of a dermoid cyst. The yellow ‘+’ symbols indicate the dimensions of the mass.

**Figure 2:**
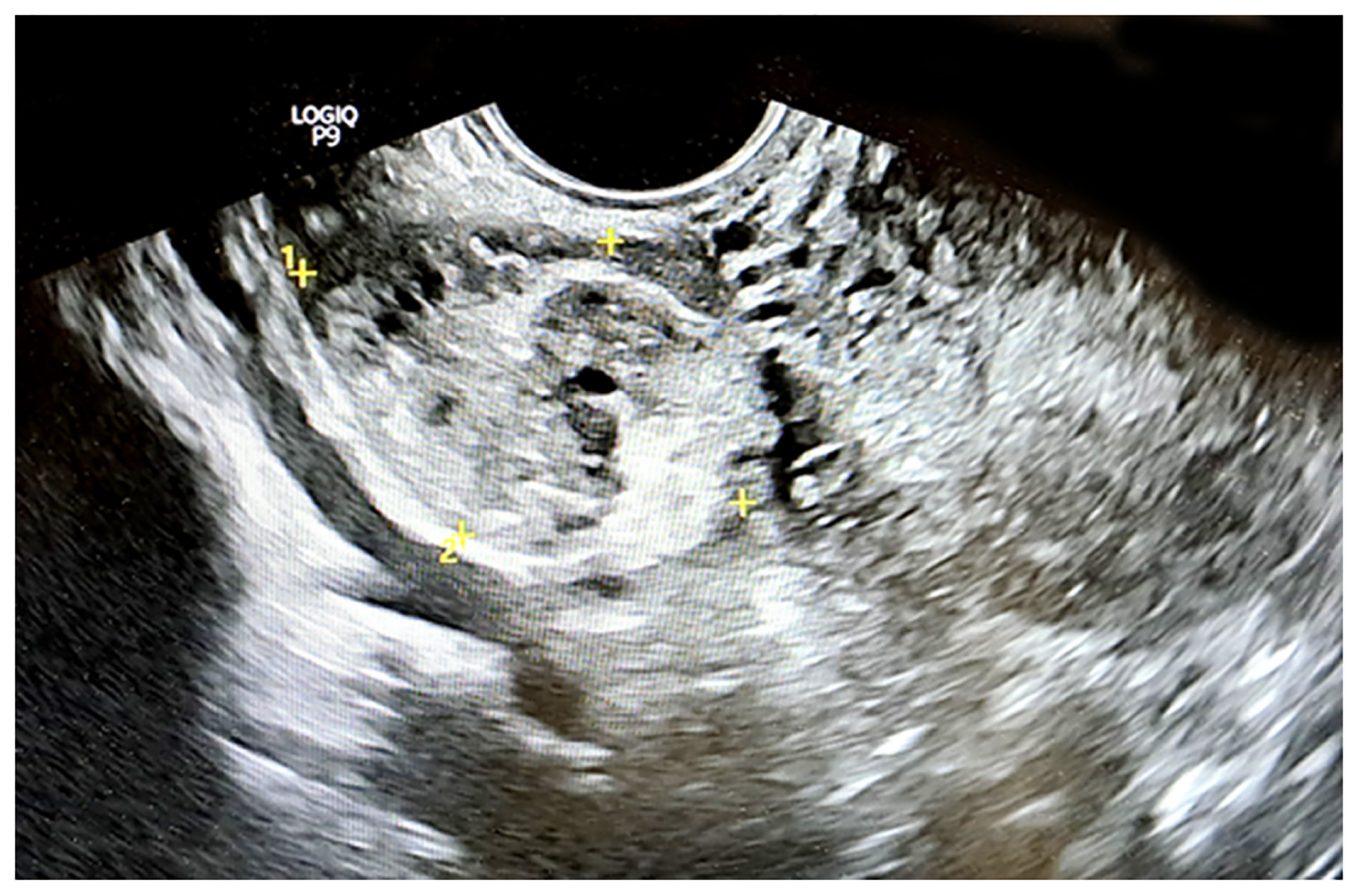
Gray-scale transvaginal ultrasonography, using cross-sectional imaging, illustrating the characteristic whirlpool sign with visualization of a twisted ovarian vascular pedicle in a plane perpendicular to the axis of torsion. The twisted vascular pedicle is identified between the right uterine cornu and the mass, measuring 3.99x2.74 cm in cross section. The yellow ‘+’ symbols indicate the dimensions of the mass.

**Figure 3:**
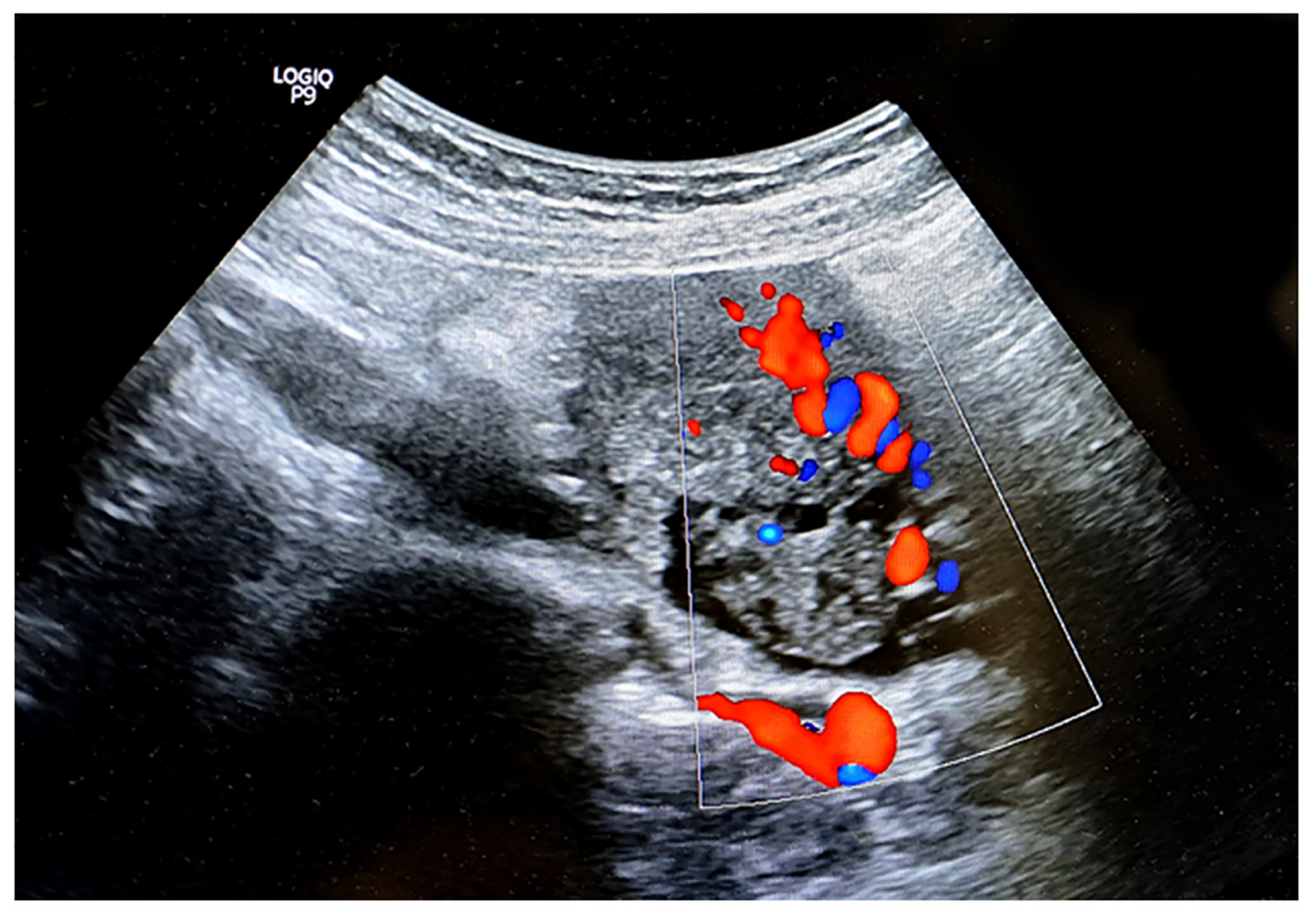
Pelvic color Doppler ultrasound illustrating the characteristic spiral ‘whirlpool sign’, highlighting torsion of the ovarian vascular pedicle.

A transvaginal ultrasonography of the right ovary, which measured 6.13x5.20 cm, identified two masses highly suggestive of mature cystic teratomas. The first mass, measuring 3.01x2.75 cm, exhibited hyperechoic foci indicative of fat and hair, whereas the second mass, measuring 3.12x2.45 cm, displayed a hyperechoic region with cystic components. Both findings were characteristic of two cystic teratomas. The left ovary appeared normal in size and echotexture (Fig. 1). The characteristic whirlpool sign was observed on the right, indicating torsion of the right adnexal vessels, thereby confirming the diagnosis of adnexal torsion (Fig. 2). Doppler flow was detected within the twisted vascular pedicle (Fig. 3). The examination of the liver, spleen, appendix and bowel did not reveal any notable findings, and no free fluid was noted in the pelvic cavity.

Laboratory test results were as follows: White blood cell count, 6.90 K/µl; hematocrit, 32.6%; hemoglobin level, 10.3 g/dl; platelet count, 239 K/µl; neutrophils, 66.4%; serum glucose, 94 mg/dl; urea, 9 mg/dl; creatinine, 0.5 mg/dl; and C-reactive protein, 0.04 mg/dl (reference range, 0-0.5 mg/dl). Serum glutamic-oxaloacetic transaminase (14 IU/l), serum glutamic-pyruvic transaminase (11 IU/l) and serum electrolyte levels (potassium, 3.6 mmol/l; sodium, 137 mmol/l) were within normal reference ranges. A complete urinalysis did not reveal any notable findings.

**Figure 4:**
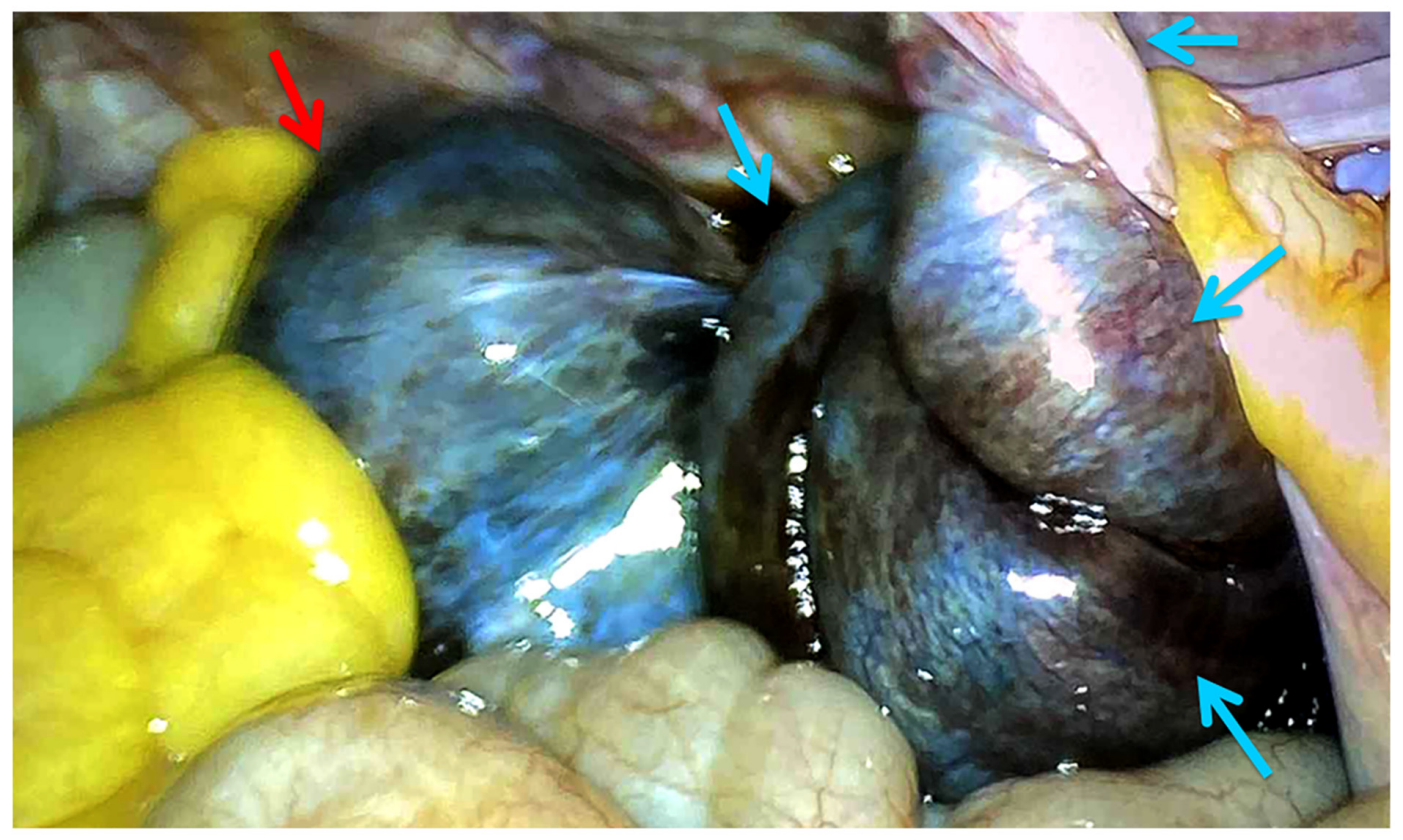
Intraoperative image illustrating a 6-cm bluish-black in color mass (red arrow) located in the posterior pouch of Douglas, twisted 1440˚ (blue arrows) around its infundibulopelvic ligament axis.

Given the acute presentation, the patient underwent an emergency laparoscopy under general anesthesia. Intraoperatively, a 6-cm right ovarian cyst was found to be quadruply twisted around its vascular pedicle (Fig. 4). The right adnexa appeared hyperemic, edematous, and bluish-black in color. The left ovary was noted to be normal. A right salpingo-oophorectomy was performed, as the patient had completed her family and did not wish to preserve fertility. No intraoperative complications occurred. The patient was monitored in the hospital for 2 days and subsequently discharged in stable condition. No complications or recurrences were observed during outpatient follow-up. Post-operative pathological examination confirmed torsion of mature ovarian teratomas with hemorrhagic necrosis.

## Discussion

Mature teratomas, commonly referred to as dermoid cysts, are among the most frequently encountered benign ovarian tumors and are bilateral in ~10-15% of cases (18). These lesions originate from totipotent germ cells and characteristically can contain tissues derived from all three embryonic germ layers (18). Their contents typically include sebaceous material and hair, while the cyst wall is partially lined by keratinized squamous epithelium containing hair follicles and sebaceous glands (18). Additional well-differentiated tissues, including teeth, bone, cartilage, thyroid tissue and respiratory-type epithelium, may be identified within these lesions (18). They have fatty components, with or without associated calcifications (18). Mature teratomas occur predominantly in younger patients (18). In addition, multiple synchronous ovarian teratomas may occur within the same ovary (18). The predisposition of ovarian teratomas to torsion is largely attributed to their size, weight and relative mobility within the adnexa (5,11). In particular, the presence of a large adnexal mass with heterogeneous internal contents, as is often the case with ovarian teratomas, increases the volume and mobility of the mass, thereby facilitating its rotation around its pedicle (5,11). This twisting may lead to sudden vascular compromise, resulting in acute, severe pain and often necessitating urgent surgical intervention (19). The spontaneous rupture of ovarian teratomas has been reported in ~3-7% of cases (18). Malignant transformation occurs in a small proportion of patients, with an estimated incidence of ~1.8% (18). Ovarian teratomas exhibit distinctive ultrasonographic features, most notably a markedly hyperechoic ovarian mass or a hyperechoic mural nodule, commonly referred to as a dermoid plug (20). Additional sonographic findings may include areas of calcification and fat-fluid levels, which together contribute to their characteristic imaging appearance (20). In the patient described herein, ultrasonographic findings revealed two ovarian masses highly characteristic of mature cystic teratomas within the right ovary measuring 6.13x5.20 cm. The first mass (3.01x2.75 cm) demonstrated hyperechoic foci consistent with fat and hair, while the second mass (3.12x2.45 cm) exhibited a hyperechoic area with cystic components. Both appearances are classic features of benign, complex germ cell tumors that often contain various tissues, such as hair and fat.

Ovarian torsion is defined as partial or complete rotation of the ovarian vascular pedicle around the ligamentous support structures, the infundibulopelvic and utero-ovarian ligaments (21). This rotation initially compromises venous and lymphatic outflow, leading to ovarian congestion and stromal edema (10). In more advanced cases, it may subsequently impair arterial inflow (10). If unrecognized or untreated, these vascular disturbances can progress to ischemia, infarction, and loss of ovarian viability (10). Ovarian torsion most frequently occurs in the presence of an adnexal mass, which increases the torque applied to the vascular pedicle and serves as a mechanical fulcrum for rotation on its supporting ligaments (22). Larger masses are particularly associated with this risk, amplifying the likelihood and severity of torsion (23). The clinical presentation of adnexal torsion is most commonly characterized by sudden-onset pelvic pain accompanied by nausea and vomiting. However, these features are non-specific and overlap with those of other acute conditions, such as appendicitis, pelvic inflammatory disease and non-torsed adnexal masses. Therefore, both transabdominal and transvaginal ultrasonography are routinely employed for diagnostic evaluation (23,24). Ultrasonographic findings suggestive of adnexal torsion include ovarian enlargement with stromal edema and peripheral displacement of follicles, as well as the presence of free pelvic fluid. An associated adnexal mass further supports the diagnosis, particularly when it is >5 cm in diameter and appears heterogeneous or demonstrates complex internal architecture, such as the ovarian teratomas (1,17). Color Doppler interrogation may show absent or reduced ovarian blood flow; however, this finding is not definitive, as preserved flow may be present due to the dual arterial supply of the ovary or in cases of partial or intermittent torsion (25). Consequently, early imaging guided by clinical suspicion is essential, and the identification of a large adnexal mass, particularly a mature cystic teratoma, should further heighten concern for ovarian torsion (10). Among the ultrasonographic indicators of ovarian torsion, the whirlpool sign is regarded as the most specific (26). This sign reflects the direct visualization of a twisted ovarian vascular pedicle comprising the ovarian artery and vein, appearing as a coiled or spiral structure on both gray-scale and color Doppler imaging. It has been observed in up to 90.8% of cases (11,26). In the case presented herein, a detailed ultrasonographic examination provided clear visualization of the whirlpool sign, observed as a twisted vascular pedicle adjacent to the thickened fallopian tube and the enlarged right ovary containing two mature cystic teratomas. This finding was critical in establishing the diagnosis of adnexal torsion pre-operatively, highlighting the increased torsion risk associated with multiple teratomas in a single ovary. The clear depiction of the twisted pedicle on both gray-scale and color Doppler imaging underscored the urgency of surgical intervention. The accurate identification of this sign requires careful tracing along the anticipated course of the vascular pedicle and precise transducer positioning (27). Subtle probe adjustments are also necessary to clearly delineate the twisted configuration (27). The meta-analysis by Adu-Bredi *et al* (28) reported that the whirlpool sign has a diagnostic sensitivity of 82% [95% confidence interval (CI), 0.78-0.86] and a specificity of 81% (95% CI, 0.70-0.90), with a positive predictive value of 93.6% and a negative predictive value of 43.8%. Despite its high diagnostic value, the visualization of the whirlpool sign can be technically difficult and is highly dependent on sonographer expertise and familiarity with pelvic vascular anatomy (14). It also requires optimal transducer angulation and often a transvaginal approach to maximize spatial resolution (14). Although systematic scanning techniques that trace the vascular pedicle may improve detection rates, even experienced practitioners may fail to visualize the sign in some cases. Patient-related factors, such as body habitus and ovarian position, may further limit detection. Accordingly, standardized scanning protocols and focused training in acute gynecological ultrasound are essential. Notably, the absence of the whirlpool sign does not exclude torsion, particularly in early, partial, or intermittent cases. Imaging findings should always be interpreted in conjunction with clinical suspicion, as torsion remains a clinical diagnosis supported by imaging rather than defined by it alone. Absent ovarian Doppler flow is highly suggestive of adnexal torsion (29). However, relying on Doppler findings alone may be misleading, as arterial perfusion can persist via collateral circulation, and normal flow does not reliably exclude torsion (16,25). Therefore, a combined morphological and flow-based assessment is recommended (16,25). A standardized ultrasonographic protocol for tracing the adnexal vascular pedicle may help reduce operator dependency and improve the detection of the whirlpool sign, particularly among less experienced sonographers. The following stepwise approach can be incorporated into routine pelvic ultrasonography: The process should first begin with the systematic identification of the uterus and both ovaries using transvaginal ultrasonography, as this approach provides superior spatial resolution (10,30). Once the affected ovary is identified, typically enlarged and often associated with a mass, color or power Doppler with low velocity settings should be activated to optimize detection of slow venous flow (31,32). Subsequently, the utero-ovarian ligament should be located medially by placing the probe in the sagittal plane at the uterine cornu and gently sweeping laterally toward the ovary. This should be followed by the identification of the infundibulopelvic ligament laterally, near the pelvic sidewall, where the ovarian vessels originate. The vascular pedicle lies between these two landmarks (14,33). Using slow, deliberate probe movements (rocking, tilting and rotation), the vessels should be traced continuously from the uterus toward the adnexa, maintaining visualization of the vascular pathway rather than jumping between structures. If the pedicle is not immediately visualized, switching between transverse and oblique planes would be required, as the twisted configuration may only become apparent in specific angles (26,33). When a suspicious coiled structure is identified, color Doppler should be applied to confirm concentric or spiral flow, characteristic of the whirlpool sign (26,31). Gentle graded compression may help displace bowel loops and improve visualization (34). Finally, bilateral comparison should be performed and cine loops should be documented to capture dynamic vascular patterns. This structured tracing technique, emphasizing anatomical landmarks, continuous vessel tracking and multiplanar assessment, has been shown to improve diagnostic confidence and may increase the detection rates of adnexal torsion (14,24,26,27).

In younger patients and those desiring future fertility, the pre-operative identification of the whirlpool sign has critical implications for surgical decision-making, particularly in supporting a conservative approach with detorsion rather than oophorectomy. Visualization of the whirlpool sign confirms the presence of adnexal torsion at an early stage, often before irreversible ischemic damage has occurred (26,27,31). This early and specific diagnosis facilitates prompt surgical intervention, which is a critical determinant of ovarian salvage (30,35). Of note, multiple studies have demonstrated that even ovaries with a grossly ischemic or cyanotic appearance at laparoscopy may regain function following detorsion, supporting a paradigm shift toward ovarian conservation whenever feasible (36-38). In this context, the whirlpool sign serves as a direct imaging marker of mechanical torsion rather than tissue nonviability, thereby reinforcing the rationale for detorsion as the initial surgical step in women of reproductive age (32). Furthermore, its identification may reduce diagnostic uncertainty and avoid delays that could otherwise lead to infarction and necessitate oophorectomy (14). While the definitive intraoperative assessment of ovarian viability remains essential, the pre-operative detection of the whirlpool sign contributes to surgical planning, patient counseling and the prioritization of fertility-preserving strategies. Consequently, incorporating systematic evaluation for the whirlpool sign into ultrasound protocols may increase the likelihood of conservative management and improve reproductive outcomes in appropriate patients (35,37,39).

Recent advances in ultrasonographic technology, including three-dimensional (3D) volumetric imaging and advanced Doppler techniques, may help overcome several of the technical limitations associated with visualization of the whirlpool sign. Conventional two-dimensional ultrasonography is inherently operator-dependent and may fail to capture the complex spatial configuration of a twisted vascular pedicle, particularly when its orientation is oblique or obscured by adjacent structures. By contrast, 3D volumetric imaging enables the acquisition of a complete dataset of the adnexal region, allowing multi-planar reconstruction and post-processing analysis. This facilitates improved visualization of the spatial relationship between the ovary, fallopian tube and vascular pedicle, potentially enhancing the detection of the spiral configuration characteristic of torsion (13,40,41). Additionally, 3D power Doppler can provide a more comprehensive depiction of vascular architecture and flow distribution, even in low-flow states, thereby increasing sensitivity in early or partial torsion and enabling objective assessment of reperfusion following detorsion (40,42). Advanced Doppler modalities further improve diagnostic performance by addressing the limitations of conventional color Doppler. Research has demonstrated that abnormalities in ovarian venous flow may be detected even when arterial flow is preserved, highlighting the importance of sensitive Doppler techniques in early torsion (43). Moreover, combined gray-scale and Doppler assessment allows the stratification of torsion severity and may assist in predicting ovarian viability (42,17). These techniques may reduce false-negative findings in cases with preserved arterial flow by better characterizing venous congestion and microvascular compromise, which are early pathophysiologic events. Despite these advantages, current evidence suggests that ultrasound performance remains variable and dependent on technique, reinforcing the need for multimodal optimization and operator training (14,44). Although advanced technologies, such as high-definition flow and microvascular imaging are not yet universally available in emergency settings, they hold significant promise for improving diagnostic confidence, particularly in technically challenging or equivocal cases. Future prospective studies are required to validate their clinical utility and to establish standardized protocols for integrating 3D volumetric Doppler reconstruction and superb microvascular imaging into routine evaluation of suspected adnexal torsion (45-48). When ultrasonographic findings are atypical or inconclusive, adjunct cross-sectional imaging with computed tomography (CT) or magnetic resonance imaging (MRI) may provide additional diagnostic information. A CT scan can demonstrate twisted vascular pedicles, adnexal displacement and secondary signs of ischemia, while an MRI provides superior soft-tissue contrast for delineating the twisted pedicle and associated features (49-57). However, these modalities should be regarded as complementary to, rather than replacements for, ultrasonography in the evaluation of suspected ovarian torsion (10). A comparison of the roles of ultrasonography, CT and MRI in cases of suspected ovarian torsion is presented in Table I.

In summary, the occurrence of two or more mature cystic teratomas within a single ovary is exceedingly rare and presents unique diagnostic and clinical considerations (18,58). However, the presence of multiple or complex masses increases the overall volume and mobility of the ovary, significantly raising the risk of torsion due to enhanced rotational potential around the vascular pedicle (59,60). In addition, ovarian torsion is more likely to occur with benign ovarian tumors, particularly cystic masses such as teratomas (61). The identification of a definitive whirlpool sign on ultrasonography should prompt urgent gynecological consultation and consideration for laparoscopy, which remains the gold standard for both definitive diagnosis and management. Timely surgical intervention is strongly associated with improved rates of ovarian salvage. Clinicians and sonographers need to maintain a high index of suspicion in such cases, as prompt diagnosis and timely intervention are essential to prevent irreversible ischemic damage and to preserve ovarian function, particularly in women desiring future fertility. Published surgical series indicate that ovarian salvage is highly time-dependent, with preservation rates >90% when detorsion is performed early following symptom onset, and declining substantially with diagnostic and therapeutic delay due to progressive ischemia and hemorrhagic infarction. In cases with prolonged torsion, salvage rates may decrease by almost half, reflecting the development of irreversible necrosis (30,33). Notably, evidence from pediatric and reproductive-age populations demonstrates that the intraoperative macroscopic appearance of the ovary is an unreliable indicator of viability (62-66). Even ovaries that appear dark, cyanotic, or necrotic at the time of surgery frequently recover function following detorsion, with reported recovery rates approaching 80-90% (62-66). Together, these findings underscore the importance of early intervention, while strongly supporting a conservative, ovary-sparing surgical approach whenever feasible, particularly in patients with future fertility considerations. Looking forward, the broader adoption of high-resolution ultrasonography, advanced Doppler techniques and three-dimensional volumetric imaging may further enhance visualization of twisted vascular pedicles. In parallel, large-scale prospective studies focusing specifically on teratoma-associated torsion and the whirlpool sign are warranted to refine imaging protocols and optimize clinical pathways for this time-sensitive gynecologic emergency.

In conclusion, the ultrasonographic whirlpool sign is a highly specific and clinically valuable marker for ovarian torsion, particularly in cases with multiple mature cystic teratomas within a single ovary. The clear identification of the whirlpool sign enables a rapid pre-operative diagnosis, guiding timely surgical intervention and minimizing ischemic injury. Although detection can be influenced by operator expertise and patient factors, focused assessment of the adnexal vascular pedicle during routine ultrasonography for acute pelvic pain significantly improves diagnostic accuracy. The early recognition of the whirlpool sign is essential for optimizing patient outcomes, preserving ovarian function, particularly in women seeking fertility preservation and reducing morbidity.

## Figures and Tables

**Table I:** Comparative role of ultrasonography, CT and MRI in suspected ovarian torsion.

Imaging modality	When to prioritize	Advantages	Limitations	Typical findings in adnexal torsion	(Refs.)
Ultrasonography (US) (first-line)	Initial evaluation of all suspected cases, particularly in women of reproductive aged	Widely available, real-time, no radiation, allows Doppler assessment and whirlpool sign visualization.	Operator-dependent; limited by body habitus, bowel gas, or atypical ovarian position.	Enlarged ovary, peripheral follicles, stromal edema, free fluid, whirlpool sign.	(30,32)
Computed tomography (CT)	When US findings are inconclusive and patient presents with acute abdomen, or when the differential diagnosis includes appendicitis, bowel pathology, or urinary causes.	Rapid, widely available in emergency settings; evaluates entire abdomen and pelvis.	Ionizing radiation; limited soft tissue characterization compared to MRI.	Twisted vascular pedicle, adnexal enlargement, uterine deviation, fat stranding, hemoperitoneum.	(22,33)
Magnetic resonance imaging (MRI)	When US findings are inconclusive and patient is stable, particularly in young or pregnant patients requiring radiation avoidance.	Soft tissue contrast; superior characterization of adnexal masses; no radiation.	Limited availability, longer acquisition time, higher cost.	Twisted pedicle (whirlpool), stromal edema, hemorrhagic infarction, follicular displacement.	(49-57)
CT vs. MRI decision	CT preferred in emergency or undifferentiated acute abdomen; MRI preferred in equivocal gynecologic cases or considerations of fertility/pregnancy.	CT: Speed and availability; MRI: Tissue detail and safety.	-	-	(49,52,56,57)

## Data Availability

The data generated in the present study may be requested from the corresponding author.
